# Ecology of emergency care in lower-tier healthcare providers in Ghana: an empirical data-driven Bayesian network analytical approach

**DOI:** 10.1007/s11739-024-03607-6

**Published:** 2024-04-29

**Authors:** Ebenezer Afrifa-Yamoah, Victor Fannam Nunfam, Bernard Agyei Kwanin, Kwasi Frimpong

**Affiliations:** 1 School of Science, Edith Cowan University, Perth, WA Australia; 2 Social Development, Takoradi Technical University, Sekondi-Takoradi, Ghana; 3 School of Arts and Humanities, Edith Cowan University, Perth, WA Australia; 4 Health Facilities Regulatory Agency, Greater Accra, Accra, Ghana; 5 School of Public Service and Governance, Ghana Institute of Management and Public Administration, Achimota, Accra, Ghana; 6 School of Medical and Health Sciences, Edith Cowan University, Perth, WA Australia

**Keywords:** Accident care, Emergency care, Emergency preparedness, Health facilities, HeFEPAT

## Abstract

The healthcare landscape in Ghana is primarily composed of lower-tier providers, which serve as the initial  point of contact for most medical emergencies. This study aimed to assess the emergency care preparedness and readiness of primary healthcare providers using a robust evaluation approach. A multicentre retrospective cross-sectional study was conducted on  460 healthcare facilities  using the standardised Health Facilities Emergency Preparedness Assessment Tool (HeFEPAT). Data were analysed via Bayesian Belief network. Emergency preparedness was associated with facility location, type, ownership, and in-charge personnel. Over 70% of facilities lacked specialised emergency/critical care personnel. Although 65% of in-charges reported protocol knowledge, only 7.8% could execute cardiopulmonary resuscitation. 90% of facilities lacked onsite defibrillators, and over 80% had no cerebrovascular accident medications. Road traffic accident protocols were largely unavailable, with an estimated 53% probability of  lacking such protocols. Private-owned facilities were more likely to lack protocols for road traffic accidents (76% vs 20% probability) and general acute care (62% vs 32%) compared to government-owned facilities. Significant gaps in emergency preparedness were identified across the studied health facilities, indicating limited capacity to manage critical situations effectively. Urgent investments in emergency medicine training, essential resources, and evidence-based protocols are needed. Standardised emergency preparedness assessments should be implemented for accreditation and quality improvement. Further research can inform the development of national guidelines and targeted interventions to strengthen emergency response capacities.

**Supplementary Information:**

The online version contains supplementary material available at 10.1007/s11739-024-03607-6.

## Introduction

The growing incidence of acute life-threatening, injuries, and other health emergencies has led to a greater emphasis on the field of emergency medicine (EM). Emergency events require skilled professionals who must work swiftly within a narrow time frame that often determines the difference between life and death. In low- and middle-income countries, it has been estimated that nearly half of all deaths and one-third of disabilities could have been prevented with appropriate emergency care [1]. The wide range of diseases and conditions handled by the emergency medical system underscores the need to prioritise resources for this sector. For instance, evidence shows that malaria alone accounts for over 500 million acute cases worldwide, resulting in more than 1 million deaths, primarily in sub-Saharan Africa. Additionally, there are over 4.4 million deaths each year globally from injuries such as those caused by road traffic accidents, falls, drowning, burns, poisoning and acts of violence. These injuries represent 8% of all deaths [2]. Ironically, while most of these deaths occur in low- and middle-income countries, the average health expenditure per gross domestic product (GDP) in these countries is approximately 5%, which is significantly lower than the average of ~14% in high income countries [3]. As a result, the emergency healthcare sector in low- and middle-income countries suffers from inadequate funding, and its management is often rudimentary and ad hoc in nature, particularly in lower-tier healthcare providers such as maternity homes, health centres, clinics, polyclinics, and primary hospitals [4].

In several African countries including Ghana, the healthcare system is primarily composed of lower-tier providers, which are often the initial point of contact for medical emergencies. Over the past three decades, Ghana has experienced various major disasters, including a catastrophic stadium incident, recurrent flooding, fire disasters and gas explosions. Prior to 2001, there was no structured emergency response system in place and these providers lacked formalised medical emergency setups. In response to the stadium disaster, the national ambulance service was established in 2004 to enhance the prehospital emergency response system and improve the emergency healthcare sector. However, the government has not consistently allocated sufficient resources to support the emergency health sector. For example, in 2020, Ghana’s health expenditure as a percentage of GDP was approximately 3.99%, well below the global average of 10.89% [5]. This lack of investment extends to emergency healthcare services, resulting in an ill-resourced and deteriorating medical emergency system. 

Meanwhile, Ghana also faces significant challenges with road traffic accident fatalities, ranking among the top 10 causes of death and contributing to an estimated loss of around 8% of GDP [6–9]. Additionally, the country faces a growing  burden of both communicable and non-communicable diseases, which contribute to a substantial number of deaths.  For instance, in 2019, communicable diseases such as respiratory infections, malaria, and diarrhoeal diseases accounted for 45% of deaths, while non-communicable diseases, including stroke, ischemic heart disease, diabetes mellitus, and hypertensive heart disease, were responsible for 49% of deaths  [10]. Insufficient resources, both financial and human,  coupled with inadequate healthcare infrastructure, and a lack of emergency setups and preparedness, exacerbate the severity of most emergency cases [11]. In 2021, the healthcare workforce demographics revealed a doctor-to-patients ratio of 1:5000 and a nurse (and midwife)-to-patients ratio of 1:278 [5], highlighting the strain on the country's healthcare system.

It must be emphasised, however that, even with limited resources and absence of specialised personnel and care, a systematic care approach to the assessment and management of medical emergencies could save lives [12]. Emergency medical care is often perceived as expensive and reliant on high-tech interventions, leading to its low prioritisation, particularly in low-income countries. However, care approaches for emergency conditions can involve simple and cost-effective strategies, such as on-site management, triage, stabilisation, and the implementation of emergency response protocols [13]. In 2011, the Ministry of Health in Ghana developed a national policy on accident and emergency to ensure that health facilities have the necessary medical supplies and protocols to respond effectively to emergencies. These emergency response plans encompass a range of measures, including mobilising human resources, trained response teams, and infrastructure to prevent or effectively respond to life-threatening situations. Proper triaging, stabilisation, and prioritisation of patients based on severity and available resources are vital components of these plans. However, little attention has been given to evaluating the emergency preparedness and readiness of primary healthcare providers in Ghana. Additionally, there is a limited number of empirical studies that have examined the ecology of emergency care in lower-tier healthcare providers using robust techniques such as the Bayesian Belief Network modelling approach.

Considering this research gap in healthcare ecological studies, the key unanswered research question is—what is the extent of emergency care preparedness among lower-tier healthcare providers in Ghana? To address this gap, this study employed a Bayesian Belief Network modelling approach to thoroughly assess the emergency care preparedness of primary healthcare facilities across multiple regions in Ghana. The findings from this robust quantitative modelling technique would have significant implications for informing policy decisions and practical considerations regarding improving emergency response capabilities and preparedness of frontline healthcare providers. In particular, the granular insights on preparedness gaps at the primary care level can guide targeted investments and interventions by policymakers seeking to strengthen healthcare system resilience. Methodologically, this research establishes a rigorous foundation and blueprint for an expanded paradigm in ecological preparedness evaluation that combines real-world health facility data with advanced computational modelling. The Bayesian approach demonstrated here can be adapted and scaled up in future studies to develop predictive models and diagnostic tools that enable continuous monitoring, benchmarking, and improvement of emergency care management programs nationally. In summary, by generating actionable evidence on current deficiencies in healthcare emergency preparedness and validating an advanced Bayesian modelling technique, this study makes significant contributions on both the policy front and the research methodology frontier.

## Materials and methods

### Study design and population

A cross-sectional study design was employed, involving multiple healthcare facilities, to assess emergency care preparedness of lower-tier healthcare providers in Ghana. Data collection took place between May and August 2020, using a standardised Health Facilities Emergency Preparedness Assessment Tool (HeFEPAT). The study focused on primary health facilities registered by the Health Facilities Regulatory Agency (HeFRA) in the Greater Accra and Ashanti regions of Ghana. The list of health facilities in these regions was obtained from HeFRA, and the facilities were categorised as clinics, primary hospitals, polyclinics, maternity homes, and health centres. A purposive sampling technique was used to select 460 health facilities that met the inclusion criteria. The choice of these two regions was based on their representation of 42% of all health facilities in Ghana's 16 regions. Regional and teaching hospitals, which serve as referral centres for lower-tier facilities and have accident and emergency departments, were excluded from the study.

### Data collection instrument

The data collection instrument used in this study was the HeFEPAT, which can be found in Appendix S1 (of supplementary material). The tool was specifically designed to evaluate the availability of guidelines/Standard Operating Procedures (SOPs), appropriately trained personnel, and essential equipment required to address both medical and traumatic emergencies. The selection of medical and traumatic conditions for assessment was based on their significant impact on the global disease burden.

The assessment items were measured using a dichotomous scale, where 0 represented 'No' and 1 represented 'Yes'. Additionally, the knowledge of Cardiopulmonary Resuscitation (CPR) was measured on a four-level scale: 0 for 'non-available', 1 for 'incomplete/fail (1%-49%)', 2 for 'partially complete/pass (50%-99%)', and 3 for 'complete (100%)'. 

Furthermore, additional information was collected regarding the demography of healthcare providers, including the type of facility ownership (e.g., public, private, CHAG, NGO, etc.) and the qualifications of the person-in-charge (e.g., medical doctor, midwife, physician assistant, nurse).

### Ethical consideration

The study adhered to ethical guidelines and obtained ethical approval from the Ghana Health Service Ethics Review Committee. Prior to the commencement of the study, clearance was also obtained from the management of the health facilities included in the assessment. Study procedures, confidentiality measures, and privacy concerns were clearly explained to all participants in a language they understood. Participants were also assured of their right to withdraw from the study at any stage or time without any impact on the care they receive at the facility. The participants were further informed that the findings of the study would be used solely for academic purposes and would not be disclosed to any third party. Therefore, their anonymity was ensured throughout the study. To maintain data security, completed questionnaires and study documents were securely stored under lock and key, and computer files were password-protected to prevent unauthorised access.

### Statistical methodology and data analysis

#### Bayesian network analysis

**Fig. 1:**
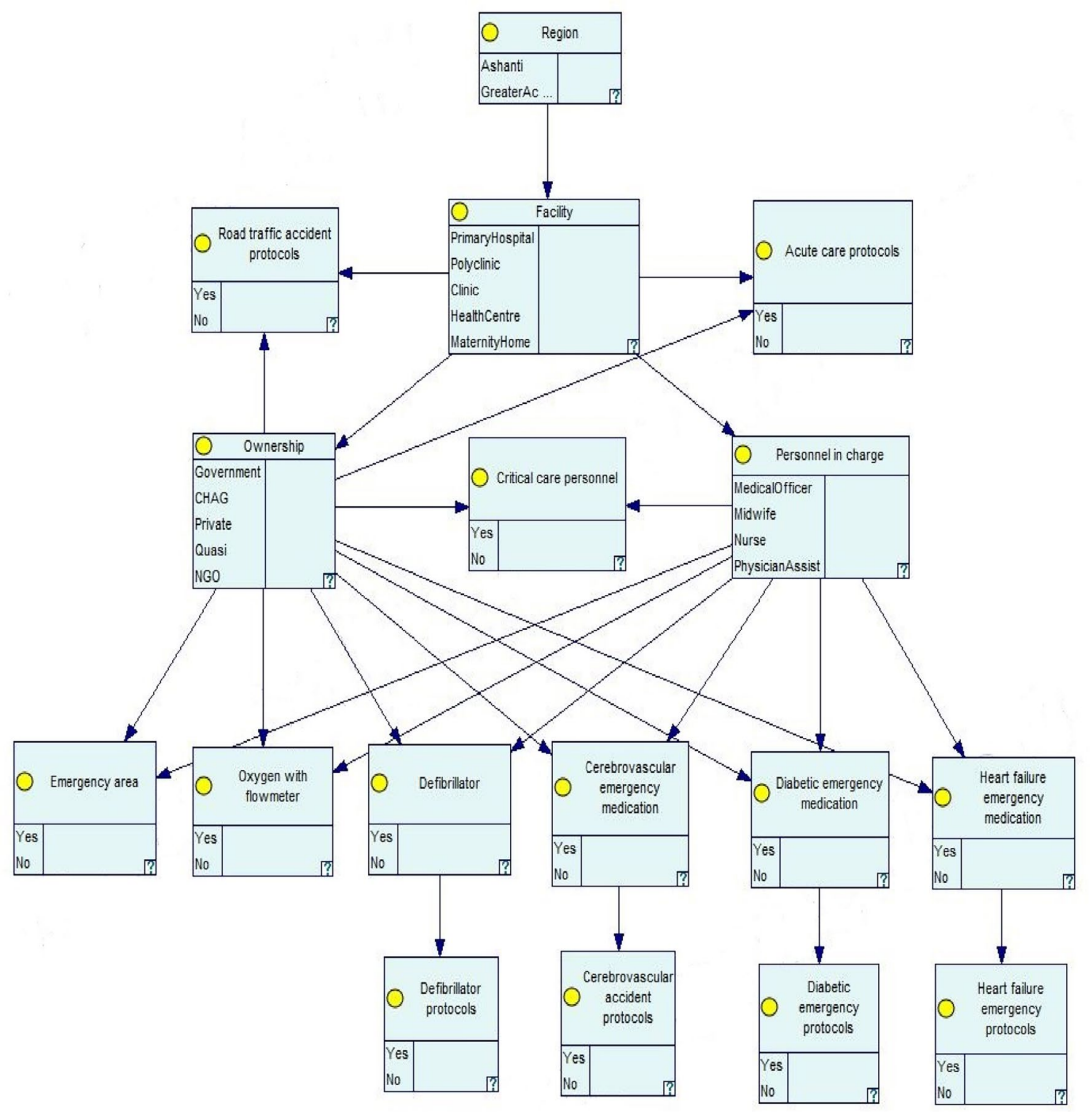
Schematic causal network for evaluating the ecology of emergency care

Bayesian network (BN) analytical approach has diverse areas of application in prediction, and decision making [14]. The class of models in Bayesian networks are useful in learning causal effects and provide intuitive graphical representation, which provides qualitative understanding of different pathways. BN is a directed acyclic graph in which nodes represent variables and edges measure the existence of causal dependence between linked variables. It provides opportunity to perform complex hierarchical evaluation of phenomenon of interest. The Bayesian network applied was developed using the software product GeNIe 4.0 [15]. The network contains 13 sub-models (see details in Table 1), with the probability factors presented in Appendix S2 (of supplementary material). The states in each chance nodes can be inferred from Fig. 1. For example, there were five states (i.e., primary hospital, or polyclinic, or clinic, or health centre, or maternity home) for facility, whilst emergency area has 2 states (i.e., yes or no). In this study, the prior probability distributions were estimated from collected data.

**Table 1:**
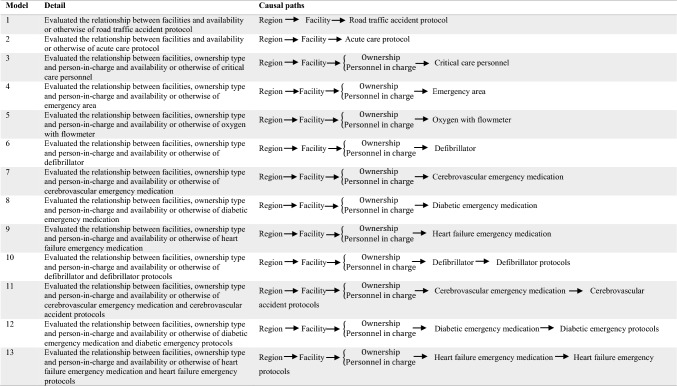
Essential causes and model structures

BNs apply Bayes’ rule, where a prior probability represents the likelihood that an input parameter will be in a particular state. Then the conditional probability calculates the likelihood of the state of a parameter given the states of input parameters affecting it; and the posterior probability is the likelihood that parameter will be in a particular state, given the input parameters, the conditional probabilities, and the rules governing how the probabilities combine. Supposed ${N}_{1}$ and ${N}_{2}$ are two connecting nodes, the network is solved when these nodes have been updated using Bayes’ rule.

1 $$P\left( {N_1 {|}N_2 } \right) = P\left( {N_2 {|}N_1 } \right)P(N_1 )/P(N_2 )$$

where $P({N}_{1})$ is the prior distribution of parameter ${N}_{1}$; $P\left({N}_{1}|{N}_{2}\right)$ is the posterior distribution, and $P\left({N}_{2}|{N}_{1}\right)$ is the likelihood function. A detailed introduction to Bayesian networks can be found in literature [14, 16, 17].

## Results

### Demographics of health facilities

The study assessed 460 health facilities across two regions in Ghana: Ashanti (*n* = 254) and Greater Accra (*n* = 206). These are the two most populous regions in the country, with populations of 4.7 million and 4 million, respectively [3]. Most facilities surveyed were primary hospitals (55.0%, 253/460), while polyclinics were the least common (1.1%, 5/460). In terms of ownership, most facilities (75.2%, 346/460) were privately owned, followed by government ownership (24.6%, 113/460), with NGOs owning the least (0.2%, 1/460). We interviewed 460 persons in charge at the health facilities. Among these respondents, the majority (72.8%, 335/460) were medical officers, followed by midwives (16.3%, 75/460), and physician assistants (10.7%, 49/460). Only one facility was managed by a nurse (see Table 2).

**Table 2:** General characteristics of health facilities

Variable	Frequency (*n*)	Percentage (*%*)
Type of Facility		
Clinic	84	18.3
Health Centre (HC)	59	12.8
Maternity Home (MH)	59	12.8
Polyclinic (Poly)	5	1.1
Primary hospital (PH)	253	55.0
Type of ownership		
CHAG	21	4.6
Government (Gov)	89	19.3
NGO	1	0.2
Private	346	75.2
Quasi	3	0.7
Location of facility		
Greater Accra	206	44.8
Ashanti	254	55.2
Person In-Charge		
Medical Officer (MO)	335	72.8
Midwife (MW)	75	16.3
Nurse (N)	1	0.2
Physician Assistant (PA)	49	10.7

### Emergency resources and preparedness of health facilities

Assessment of the availability of common emergency machinery, medications, and protocols in the health facilities revealed that many of the health facilities did not have critical care personnel (78.3%, 360/460). Out of the 460 in-charges that were interviewed, 297 (65.0%) indicated they had knowledge on acute care protocols. Also, 297 (64.6%) had areas or rooms designated as emergency area. 403 (87.61%) of the facilities had oxygen with flowmeter installed. However, most of the health facilities did not have defibrillator (90.00%, 414/460) and defibrillator protocols (417/460, 90.7%). Over 80% (371/460) of health facilities reported not having cerebrovascular accident emergency medications, with over 416/460 representing 90.40% did not have any available form of cerebrovascular accident protocols. About a third (67.8%, 312/460) of the health facilities indicated that they had diabetic emergency protocols, with 65.9% (303/460) having diabetic emergency medications in stock. Out of the 460 facilities that were assessed, 417 (90.7%) did not have heart failure protocols and 392 (85.2%) did not have heart failure emergency medications. Over 98.00% (451/460) of the health facilities did not have accident emergency protocols.

The revelations from health facilities in-charged personnel when interviewed on their knowledge level on cardiopulmonary resuscitation (CPR) were interesting. 79/460 indicated that they have the requisite skills to lay the patient on their back and open their airway, however, they would call a colleague to resuscitate a patient. Only 182 out of the 460 in-charged expressed confidence and competent in checking for breathing to decide on the appropriateness to initiate CPR. Of these 182, 162 indicated that they could go further to perform the required 30 chest compression exercise. When asked about their training in performing two rescue breaths or more until patients are fully recovered, only 36 of the in-charged reported as being duly trained.

### Bayesian Belief Network for emergency preparedness in Ghana

**Fig. 2:**
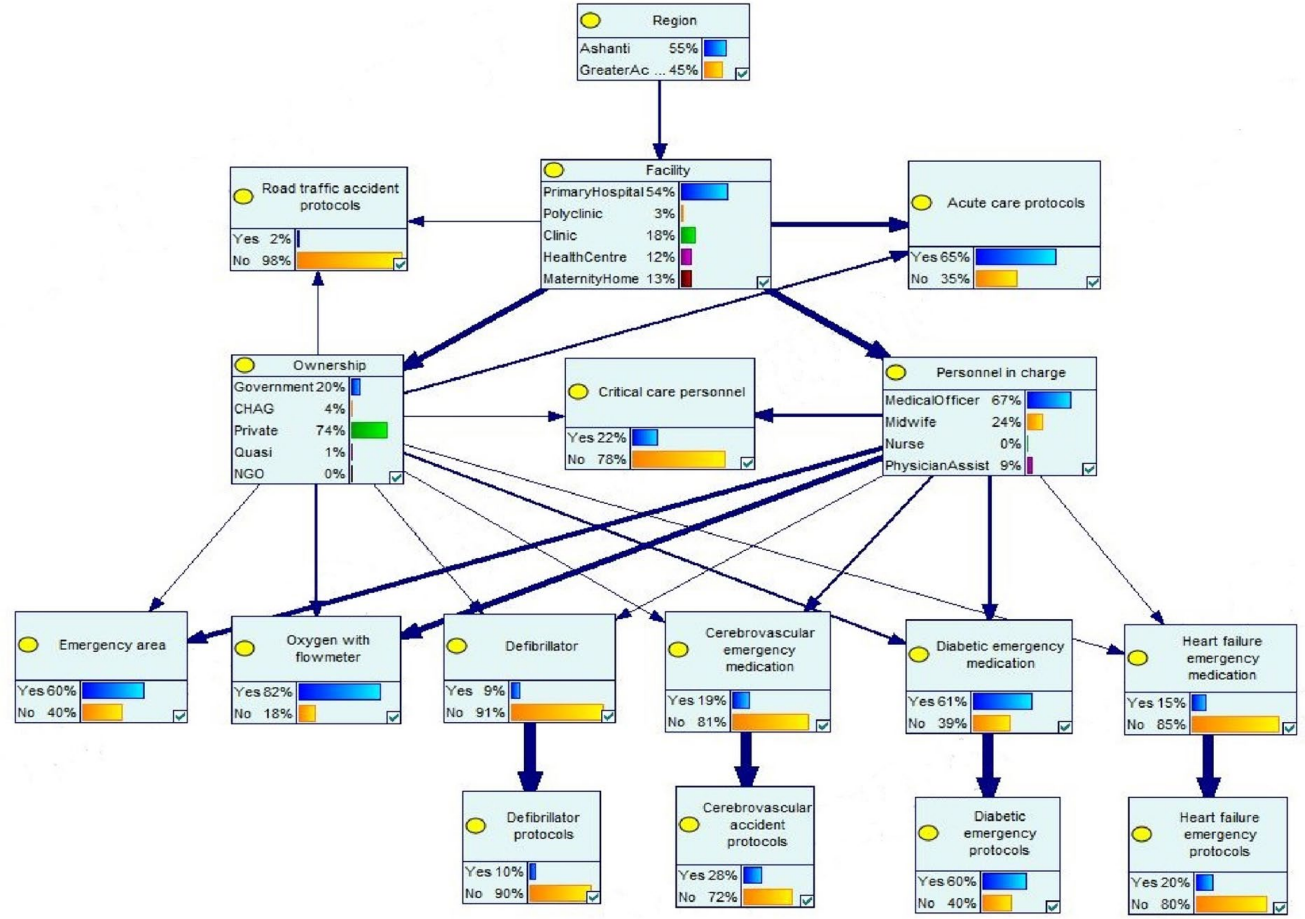
Probability distributions and sensitivity analysis of a BN model for the evaluation of the ecology of emergency healthcare in Ghana. Bold arrows indicate the strength of influence of the connecting nodes in estimating the posterior probabilities of the destination nodes

Figure 2 presents several scenarios that were modelled (see Table 1) to establish that nature of relationships that exist between key variables in the web of emergency care structure. The relative importance of the key factors that contribute to the emergency readiness of health facilities was established by assessing the change in the probability distribution of the outcome nodes, via some mediation paths.

#### Location effects

The marginal probabilities of health facilities in Ashanti and Greater Accra not having road traffic accident protocols were 0.55 and 0.45, respectively (Model 1, Table 3), indicating approximately half of the health facilities in the two populous regions do not have road traffic accident protocols help staff to manage emergency scenarios. In terms of the absence of acute care protocols, the estimated marginal probabilities for health facilities in Ashanti and Greater Accra were 0.65 and 0.31, respectively ((Model 2, Table 3). Overall, health facilities in the Greater Accra region were relatively more resourced to manage emergency scenarios compared to those in the Ashanti region.

**Table 3:** Marginal probability estimates for the key variables evaluated at the levels of the response variables for the respective models

		Model 1		Model 2		Model 3		Model 4		Model 5		Model 6		Model 7		Model 8		Model 9		Model 10		Model 11		Model 12		Model 13	
Variable	Levels	Yes	No	Yes	No	Yes	No	Yes	No	Yes	No	Yes	No	Yes	No	Yes	No	Yes	No	Yes	No	Yes	No	Yes	No	Yes	No
Region	Ashanti	0.39	0.55	0.48	0.69	0.5	0.56	0.48	0.65	0.52	0.67	0.45	0.56	0.5	0.56	0.49	0.65	0.5	0.56	0.46	0.56	0.52	0.56	0.49	0.64	0.52	0.56
	G Accra	0.61	0.45	0.52	0.31	0.5	0.44	0.52	0.35	0.48	0.33	0.55	0.44	0.5	0.44	0.51	0.35	0.5	0.44	0.54	0.44	0.48	0.44	0.51	0.36	0.48	0.44
Facility	PH	1	0.53	0.72	0.1	0.76	0.48	0.79	0.17	0.64	0.11	0.86	0.51	0.77	0.49	0.76	0.2	0.76	0.5	0.83	0.51	0.68	0.49	0.75	0.23	0.69	0.5
	Poly	0	0.03	0.05	0	0.11	0.01	0.05	0	0.04	0	0	0.03	0.1	0.01	0.05	0	0.11	0.02	0	0.03	0.07	0.01	0.05	0	0.09	0.02
	Clinic	0	0.18	0.1	0.32	0.1	0.2	0.13	0.24	0.14	0.33	0.14	0.18	0.11	0.19	0.13	0.25	0.11	0.19	0.15	0.18	0.14	0.19	0.13	0.24	0.13	0.19
	HC	0	0.13	0.02	0.3	0.02	0.15	0.01	0.29	0.13	0.1	0	0.14	0.02	0.15	0.05	0.24	0.02	0.14	0.01	0.14	0.06	0.15	0.05	0.23	0.05	0.14
	MH	0	0.13	0.05	0.27	0	0.16	0.01	0.31	0.06	0.46	0	0.14	0	0.16	0.01	0.31	0	0.15	0.01	0.14	0.05	0.16	0.02	0.3	0.04	0.15
Ownership	Gov	0.59	0.2	0.14	0.32	0.38	0.15	0.17	0.26	0.23	0.07	0	0.22	0.34	0.17	0.2	0.2	0.37	0.18	0.01	0.22	0.29	0.17	0.2	0.2	0.32	0.18
	CHAG	0	0.05	0.04	0.05	0.06	0.04	0.06	0.01	0.05	0.01	0	0.05	0.03	0.05	0.06	0.02	0.09	0.04	0	0.05	0.04	0.05	0.06	0.02	0.07	0.04
	Private	0	0.76	0.8	0.62	0.51	0.81	0.75	0.73	0.7	0.92	0.9	0.72	0.58	0.78	0.72	0.77	0.54	0.77	0.89	0.72	0.64	0.78	0.72	0.77	0.6	0.77
	Quasi	0.41	0	0.01	0	0.04	0	0.01	0	0.01	0	0.1	0	0.05	0	0.01	0	0	0.01	0.09	0	0.03	0	0.01	0	0	0.01
	NGO	0	0	0	0	0.01	0	0	0	0	0	0	0	0	0	0	0.01	0	0	0	0	0	0	0	0.01	0	0
Personnel in charge	MO	1	0.66	0.87	0.28	1	0.57	0.98	0.2	0.78	0.13	1	0.63	1	0.59	0.94	0.23	1	0.61	0.97	0.63	0.87	0.59	0.93	0.27	0.9	0.61
	MW	0	0.24	0.11	0.48	0	0.3	0.02	0.57	0.12	0.79	0	0.26	0	0.29	0.03	0.57	0	0.28	0.02	0.26	0.09	0.29	0.03	0.54	0.07	0.28
	N	0	0	0	0.01	0	0	0	0.01	0	0.01	0	0	0	0	0	0.01	0	0	0	0	0	0	0	0.01	0	0
	PA	0	0.1	0.02	0.23	0	0.12	0	0.23	0.1	0.06	0	0.1	0	0.12	0.04	0.19	0	0.11	0.01	0.1	0.04	0.12	0.04	0.18	0.03	0.11
Road traffic accident protocols	Yes	–	–	0.03	0.01	0.09	0	0.03	0	0.03	0	0.1	0.01	0.09	0.01	0.03	0	0.05	0.02	0.09	0.01	0.06	0.01	0.03	0	0.04	0.02
	No			0.97	0.99	0.91	1	0.97	1	0.97	1	0.9	0.99	0.91	0.99	0.97	1	0.95	0.98	0.91	0.99	0.94	0.99	0.97	1	0.96	0.98
Acute care protocols	Yes	0.88	0.65	–	–	0.83	0.61	0.84	0.38	0.71	0.39	0.89	0.63	0.84	0.61	0.81	0.41	0.82	0.63	0.88	0.63	0.77	0.61	0.81	0.43	0.77	0.63
	No	0.12	0.35			0.17	0.39	0.16	0.62	0.29	0.61	0.11	0.37	0.16	0.39	0.19	0.59	0.18	0.37	0.12	0.37	0.23	0.39	0.19	0.57	0.23	0.37
Critical care personnel	Yes	0.9	0.2	0.28	0.11	–	–	0.34	0.04	0.26	0.03	0.29	0.21	0.47	0.16	0.32	0.06	0.46	0.18	0.29	0.21	0.37	0.16	0.32	0.07	0.38	0.18
	No	0.1	0.8	0.72	0.89			0.66	0.96	0.74	0.97	0.71	0.79	0.53	0.84	0.68	0.94	0.54	0.82	0.71	0.79	0.63	0.84	0.68	0.93	0.62	0.82
Emergency area	Yes	1	0.59	0.77	0.28	0.92	0.51	–	–	0.7	0.16	0.86	0.58	0.91	0.53	0.83	0.23	0.92	0.55	0.84	0.58	0.79	0.53	0.83	0.26	0.82	0.55
	No	0	0.41	0.23	0.72	0.08	0.49			0.3	0.84	0.14	0.42	0.09	0.47	0.17	0.77	0.08	0.45	0.16	0.42	0.21	0.47	0.17	0.74	0.18	0.45
Oxygen with flowmeter	Yes	1	0.82	0.89	0.68	0.97	0.78	0.95	0.62	–	–	0.96	0.81	0.97	0.78	0.95	0.61	0.97	0.79	0.95	0.81	0.91	0.78	0.95	0.63	0.93	0.79
	No	0	0.18	0.11	0.032	0.03	0.22	0.05	0.38			0.04	0.19	0.03	0.22	0.05	0.39	0.03	0.21	0.05	0.19	0.09	0.22	0.05	0.37	0.07	0.21
Defibrillator	Yes	0.41	0.08	0.12	0.03	0.12	0.08	0.13	0.03	0.1	0.02	–	–	0.14	0.08	0.12	0.04	0.08	0.09	0.93	0	0.12	0.08	0.12	0.04	0.09	0.09
	No	0.59	0.92	0.88	0.97	0.88	0.92	0.87	0.97	0.9	0.98			0.86	0.92	0.88	0.96	0.92	0.91	0.07	1	0.88	0.92	0.88	0.96	0.91	0.91
Defibrillator protocols	Yes	0.42	0.09	0.13	0.03	0.12	0.09	0.13	0.04	0.11	0.03	1	0.01	0.14	0.08	0.13	0.04	0.09	0.1	–	–	0.12	0.08	0.13	0.05	0.09	0.1
	No	0.58	0.91	0.87	0.97	0.88	0.91	0.87	0.96	0.89	0.97	0	0.99	0.86	0.92	0.87	0.96	0.91	0.9			0.88	0.92	0.87	0.95	0.91	0.9
Cerebrovascular emergency medication	Yes	0.79	0.18	0.25	0.09	0.41	0.13	0.29	0.04	0.23	0.03	0.29	0.18	–	–	0.28	0.05	0.37	0.16	0.28	0.18	0.69	0	0.28	0.06	0.32	0.16
	No	0.21	0.82	0.75	0.91	0.59	0.87	0.71	0.96	0.77	0.97	0.71	0.82			0.72	0.95	0.63	0.84	0.72	0.82	0.31	1	0.72	0.94	0.68	0.84
Cerebrovascular accident protocols	Yes	0.82	0.27	0.33	0.18	0.47	0.22	0.37	0.15	0.31	0.13	0.37	0.27	1	0.11	0.36	0.15	0.44	0.25	0.36	0.27	–	–	0.36	0.16	0.39	0.25
	No	0.18	0.73	0.67	0.82	0.53	0.78	0.63	0.85	0.69	0.87	0.63	0.73	0	0.89	0.64	0.85	0.56	0.75	0.64	0.73			0.64	0.84	0.61	0.75
Diabetic emergency medication	Yes	1	0.61	0.76	0.34	0.9	0.53	0.85	0.25	0.71	0.17	0.85	0.59	0.9	0.55	–	–	0.91	0.56	0.83	0.59	0.79	0.55	0.99	0.06	0.82	0.56
	No	0	0.39	0.24	0.66	0.1	0.47	0.15	0.75	0.29	0.83	0.15	0.41	0.1	0.45			0.09	0.44	0.17	0.41	0.21	0.45	0.01	0.94	0.18	0.44
Diabetic emergency protocols	Yes	0.96	0.59	0.74	0.34	0.87	0.52	0.82	0.26	0.69	0.18	0.82	0.58	0.87	0.53	0.96	0.02	0.88	0.55	0.8	0.58	0.76	0.53	–	–	0.79	0.55
	No	0.04	0.41	0.26	0.66	0.13	0.48	0.18	0.74	0.31	0.82	0.18	0.42	0.13	0.47	0.04	0.98	0.12	0.45	0.2	0.42	0.24	0.47			0.21	0.42
Heart failure emergency medication	Yes	0.32	0.14	0.18	0.07	0.3	0.1	0.22	0.03	0.17	0.02	0.14	0.15	0.28	0.11	0.22	0.03	–	–	0.14	0.15	0.23	0.11	0.21	0.04	0.74	0
	No	0.68	0.86	0.82	0.93	0.7	0.9	0.78	0.97	0.83	0.98	0.86	0.85	0.72	0.89	0.78	0.97			0.86	0.85	0.77	0.89	0.79	0.96	0.26	1
Heart emergency protocols	Yes	0.36	0.19	0.23	0.13	0.35	0.16	0.27	0.09	0.22	0.08	0.19	0.2	0.33	0.17	0.26	0.09	1	0.06	0.19	0.2	0.28	0.17	0.26	0.1	–	–
	No	0.64	0.81	0.77	0.87	0.65	0.84	0.73	0.91	0.78	0.92	0.81	0.8	0.67	0.83	0.74	0.91	0	0.94	0.81	0.8	0.72	0.83	0.74	0.9		

#### Facility type effects

The type of facility had a significant influence on the availability of acute care protocols, but not on the availability of road traffic accident protocols. Primary hospitals, polyclinics and clinics were more likely to have the acute care protocols compared to health centres and maternity homes. Road traffic accident protocols were scarcely available in health facilities, even at the public hospitals, with a marginal probability of absence estimated at 0.53 (Model 1, Table 3). On the other hand, acute care protocols were comparatively more available in the health facilities especially in the public hospitals (with estimated marginal probability of 0.72). The majority of the lower-tier health facilities (clinics, health centres and maternity homes) were less likely to have designated emergency area space. The estimated marginal probabilities for not having emergency areas were 0.24, 0.29 and 0.34 respectively for clinics, health centres and maternity homes (Model 4, Table 3). Comparatively, it was highly less likely to have oxygen with flowmeter installed in maternity homes. The estimated marginal probability for maternity homes not having oxygen with flowmeter was 0.46, implying that almost half of the maternity homes do not have this critical instrument at site. The situation was not typically better in the clinics, with an estimated marginal probability of 0.33 not having oxygen with flowmeter. Even in the public hospitals, key emergency items such as defibrillator, cerebrovascular emergency medication and heart failure emergency medication were not readily available. For instance, the estimated marginal probabilities of public hospitals not respectively having these emergency items were 0.51, 0.49 and 0.50. The marginal probability for a public hospital not having a critical care personnel for emergency scenarios was 0.48, and this deficiency was twice more prevalent in the other facilities.

#### Ownership effects

Government owned facilities compared to private owned were more likely to have road traffic accident protocols and acute care protocols. The estimated marginal probabilities for private health facilities not having road traffic accident protocols and acute care protocols were 0.76 and 0.62, respectively, compared to government owned facilities with probabilities 0.20 and 0.32 (Model 1 & 2, Table 3). Additionally, there was significant shortage of critical care personnel in the private health facilities, with an estimated marginal probability of absence being 0.81. Comparatively, the private health facilities were relatively ill-prepared for managing emergency scenarios. For instance, there was a higher propensity to miss emergency items such as defibrillator, oxygen with flowmeter, cerebrovascular, diabetic and heart failure emergency medications in the private health facilities. The absence of these emergency kits implied the absence of their respective protocols in the health facilities. Interestingly there was greater propensity for health facilities irrespective of ownership to have no designated emergency area.

#### Personnel-in-charge effects

Personnel-in-charge was greatly influenced by the type of facility. The personnel-in-charge of the health facility significantly influenced the presence or absence of emergency areas in health facilities. The conditional probabilities of non-availability of emergency areas in health facilities managed by medical officers, midwifes, nurses and physician assistants were 0.20, 0.57, 0.01 and 0.23 respectively (Model 4, Table 3). This highlights that it is less likely to find emergency areas in maternity homes. The availability of critical care persons at the facilities was mainly influenced by the personnel-in-charge, however, the distribution of critical care persons in the health facilities was negatively skewed (22% Yes and 78% No), indicating that even most of the medical officers lacked the skills to respond to health emergency episodes. Health facilities with medical officers as in-charge were relatively more emergency prepared compared to the cases where facilities were managed by physician assistants, midwives, or nurses. For instance, the estimated marginal probability of the non-availability of oxygen with flowmeter in health facilities managed by medical officers and midwives were 0.13 and 0.79, respectively. Additionally, facilities managed by medical officers were more likely to have acute care protocols compared to facilities managed by other health professionals. Meanwhile, the availability of road traffic accident protocols in health facilities was independent of the personnel-in-charge.

## Discussion

Emergencies often present urgent threat to human wellbeing, property and/or environment. According to United Nations Office for the Coordination of Humanitarian Affairs, the scope and frequency of medical emergencies have increased nearly three times in recent times compared to about 40 years ago [18]. Most of these emergencies necessitate medical interventions to avert their immediate threat to life. The current empirical data-driven assessment of emergency care capacities across 460 health facilities in the two most populous regions (i.e., Ashanti and Greater Accra) in Ghana reveals several critical gaps that require urgent attention. The lack of adequate qualified personnel, equipment, medications, protocols, and training indicates that most facilities are ill-prepared to effectively manage medical emergencies. A key finding is the shortage of critical care staff, with 78.3% of facilities lacking specialised personnel to handle emergency and critical care. This shortage of trained emergency care professionals significantly limits these facilities' abilities to manage acute, life-threatening cases. Even where equipment and medications are available, few staff have the expertise to use them appropriately. Targeted training and recruitment of critical care providers should be prioritised.

Another major gap was in supplies and infrastructure for emergency care. While most facilities had basic oxygen, the vast majority lacked essential equipment like defibrillators and monitoring devices, along with associated protocols. Defibrillation within minutes is often essential for cardiac arrest patients, and not having these devices severely limits resuscitation capabilities. There were also shortages of lifesaving medications for conditions such as  stroke, diabetes complications, and heart failure. With 80–90% of health facilities lacking these medications and protocols, it is unlikely emergencies like diabetic crises, heart attacks, strokes, and trauma can be managed appropriately. Meanwhile, cardiovascular diseases or heart failure emergencies have emerged as a major health threat, and quite prevalent among the working class causing significant economic losses. For example, in the 2019, approximately 74% of global mortalities were attributed to non-communicable diseases. Cardiovascular related deaths accounted for about 32%, with Ischaemic heart disease and stroke being the two most fatal conditions, accounting for 16% and 11.2% respectively of the global death [2]. In sub-Saharan Africa, cardiovascular diseases-related mortalities averagely contribute to about 9.2% of all deaths [18, 19]. In Ghana, Ischaemic heart disease is the 4th leading cause of death, with incidence rate of approximately 47 deaths per 1000 population [2]. These conditions form part of the medical conditions which require immediate health attention else they will result in premature deaths. Equipping facilities with standardised emergency crash carts containing protocols, medications, and devices could help bridge these gaps efficiently. This could lead to preventable patient mortality and morbidity.

The study also revealed significant gaps in cardiopulmonary resuscitation (CPR) skills among healthcare workers. CPR can help save a life during cardiac arrest, when the heart stops beating or beats too ineffectively to circulate blood to the brain and other vital organs. However, even after training, remembering the CPR steps and administering them correctly can be a challenge. CPR training is another domain requiring urgent attention and regular training [20]. With only 36 out of 460 in-charges adequately trained in CPR, most facilities lack staff who are competent in this basic lifesaving technique. Routine training and skills assessments should be implemented based on international CPR guidelines. These could be augmented with brief intermittent CPR training for all clinical staff to help increase competency levels. Having properly trained staff is essential for administering appropriate emergency protocols, operating specialised equipment, and providing lifesaving interventions during time-sensitive emergencies.

The absence of standardised protocols compromises the capacity to rapidly mobilise staff, allocate resources, and provide coordinated care. Specifically, the lack of protocols for two common medical emergencies, namely, road traffic accident and acute care protocols, highlights systemic inadequacies in these facilities' abilities to effectively manage such scenarios. We observed a location effect on the severity of inadequacy. There were higher probabilities for health facilities in the Ashanti region to lack road accident protocols (55%) and acute care protocols (65%) compared to facilities in the Greater Accra region with probabilities of 45% and 31% respectively. As the most urbanised region, containing the national capital Accra, Greater Accra having more emergency care resources is predictable. Still, nearly a third of facilities lacking acute care protocols is concerning given the population density and health risks in Accra. Overall, the regional disparity points to unequal distribution of emergency preparedness across Ghana's health system. There is a clear need for comprehensive assessments of emergency protocols and capacities in facilities across all regions [21]. A well-balanced regional health system must be urgently prioritised to strengthen emergency response and ultimately protect patient outcomes.

The type of facility significantly influenced the availability of protocols and resources for emergency care, which highlight important disparity in emergency and acute care preparedness across different lower-tier healthcare facilities in the country. Primary hospitals, polyclinics, and clinics were more likely to have acute care protocols compared to health centres and maternity homes. This aligns with previous research showing that hospitals and larger facilities generally have more standardised policies and procedures for emergency situations [22]. However, even at the hospital-level, availability of protocols specifically for road traffic accidents was relatively low, with just a 53% probability of having them on hand. This lack of trauma-specific protocols is concerning given the high burden of injuries from road crashes in many developing countries [7, 10, 23]. In Ghana, mortalities and permanent bodily injuries associated with road traffic accidents are alarming and a major public health issue. For example, between January and October of 2020, 12,096 road traffic accidents involving over 20,400 vehicles were recorded [24]. It is reported that 72 persons out of every 100 000 population, suffered from grievous bodily injury, and close to eight of the same population died from road traffic accidents over the past decade [8, 25]. Recent WHO guidelines have called for improved trauma and injury protocols across all levels of healthcare facilities globally [26]. In medical crises, preparedness saves lives. The lack of protocols would lead to disorganisation and delay in care, risking preventable morbidity and mortality.

The availability of emergency equipment and infrastructure was predictably worse in clinics and maternity homes compared to primary hospital and polyclinics. For example, oxygen with flowmeters were missing in an estimated 46% of maternity homes. Oxygen is considered an essential medicine by the WHO and a lifesaving intervention for many childbirth and neonatal emergencies [26, 27]. Its absence in lower-level facilities likely indicates a lack of basic emergency obstetric capacity. Similar gaps have been reported in maternal health facilities globally, contributing to preventable mortality in obstetric and neonatal emergencies [28]. Even at the hospital-level, life-saving resources such as defibrillators, stroke medications, and heart failure medications were often absent. This aligns with prior studies showing major gaps in basic emergency equipment and medications in many developing country hospitals [22]. The lack of trained critical care personnel further compounds these resource limitations.

The study found important differences in emergency preparedness between government-owned and private-owned health facilities. A major deficiency across both facility types was a lack of designated emergency area space, highlighting the infrastructure limitations faced even by government hospitals in many developing nations. However, the government-owned facilities were relatively more likely to have protocols and critical resources for emergency and trauma care compared to private-owned facilities. Specifically, government facilities had a higher probability of having road traffic accident and acute care protocols. They also had significantly greater availability of trained emergency personnel. Additionally, life-saving equipment like defibrillators and oxygen tanks were more commonly present in government facilities. These findings align with prior research demonstrating gaps in emergency care capacity at private facilities in low- and middle-income countries [29, 30]. The absence of these basic protocols and resources in many private facilities could be attributed to a lack of oversight, and financial and infrastructural limitations. However, improving private sector emergency preparedness is essential given increasing privatisation of health systems globally [30]. Stronger regulation and oversight of minimum standards for protocols, staffing, medications, and equipment may help bridge emergency care disparities across different facility ownerships. Ultimately, integrating private facilities into coordinated trauma systems and emergency care networks could strengthen capacity at all levels of the healthcare system [30].

The data indicate that the qualifications and training of the personnel-in-charge have a significant influence on the emergency care capacity at health facilities. Facilities managed by medical doctors appeared to be generally better equipped and prepared compared to those managed by midwives, nurses, or physician assistants. Having a medical doctor as in-charge increased the likelihood of having dedicated emergency areas and critical care staff. This suggests doctors are better able to advocate for and provide oversight of emergency resources. The lack of emergency areas in 57% of midwife-managed facilities is particularly concerning given their role in obstetric emergencies. For instance, a multi-centre prospective cross-sectional study found that preeclampsia, which is a hypertensive disorder of pregnancy (HDP) and a major health burden in the obstetric population, is highly prevalent (8.8%) in Ghana [31]. Ensuring midwives receive leadership and emergency care training could help address this gap.

## Policy implications and recommendations

To address the gaps identified in emergency care capacity, a coordinated effort is needed to improve compliance, attract specialised staff, and supply critical materials. Consequently, this study has provided several significant policy implications and recommendations as follows.

First, our study underscores the need for government health authorities to mandate the implementation of standardised emergency care protocols across all healthcare facilities. These protocols should cover procedures for managing common medical emergencies such as cardiac arrest, stroke, and trauma. This has the tendency of ensuring adherence to standardized protocols which can improve the quality and consistency of emergency care delivery.

Second, this study acknowledges the importance of policymakers to invest in comprehensive training programmes for healthcare professionals, which focus on emergency medicine and critical care. This includes incentivising specialised training, offering continuous education opportunities, and integrating emergency care training into existing healthcare curricula. Thus, by enhancing the skills and knowledge of healthcare providers, the quality of emergency care can be significantly improved.

Third, the policy implications of this study focus on the need for government agencies and healthcare institutions to develop targeted recruitment and retention strategies that attract and retain specialised personnel in emergency care. This may include offering competitive salaries, providing career advancement opportunities, and creating supportive work environments for emergency care professionals.

Fourth, our study highlights the significance of policymakers in prioritising infrastructure development and resource allocation for emergency care facilities. This includes ensuring the availability of essential equipment, medications, and supplies for managing medical emergencies. In addition, investments should be made in upgrading infrastructure to support emergency care delivery, such as the provision of dedicated emergency rooms and ambulance services.

Fifth, the policy ramifications of our study accentuate the importance of government health authorities establishing robust systems for monitoring and evaluating the quality of emergency care services. This involves conducting regular audits, performance evaluations, and patient satisfaction surveys to assess the effectiveness of emergency care delivery. Thus, actionable feedback from these evaluations can inform targeted interventions to address gaps and improve service quality.

Sixth, policy decision makers can be informed by this study to explore opportunities for public–private partnerships to strengthen emergency care services. This may involve collaborating with private healthcare providers to expand access to emergency care facilities, leveraging private sector resources for infrastructure development, and fostering knowledge exchange and capacity building initiatives.

Seventh, this study underlies the need for government legislation to be enacted or strengthened to support the implementation of emergency care policies and regulations. This includes establishing legal frameworks for emergency medical services, which ensure compliance with quality standards, and enforcing accountability mechanisms for healthcare facilities that fail to meet emergency care requirements.

Finally, our study emphasises the policy significance of directing efforts at engaging communities and raising awareness about the importance of emergency care preparedness. This can be achieved through public education campaigns, community outreach programmes, and the establishment of community-based emergency response teams. By empowering communities to recognise and respond to medical emergencies we are contributing to improving patient outcomes and reducing morbidity and mortality rates.

Thus, by implementing these policy implications and recommendations, policymakers and healthcare practitioners can contribute to strengthening emergency care systems, enhancing patient outcomes, and ultimately improving public health in Ghana.

## Limitations

This study has inherent limitations that warrant consideration when interpreting the results. While offering valuable insights, our focus on 460 healthcare facilities in two regions (Ashanti and Greater Accra) may raise questions about generalisability beyond the sampled locations. Though these regions contain over 40% of Ghana's population and most of healthcare facilities, the sample represents only 16% of national facilities. To help assess representativeness, we compare sample demographics and outcomes to available regional and national benchmarks. We acknowledge potential sampling and response biases, and have detailed our survey recruitment methods, and sample characteristics to support transparency. A larger multi-region study could improve generalisability across diverse settings in Ghana. Additionally, our focus on specific emergency care aspects may overlook nuances in communication systems, patient outcomes, and external emergency service coordination. A more comprehensive set of variables would enable fuller understanding of emergency preparedness.

## Conclusion

We have highlighted major gaps in emergency care capacity across personnel, equipment, medications, protocols, training, and quality processes. A systematic, multifaceted response is required to upgrade the ability of health facilities to effectively manage medical crises. Having robust emergency healthcare systems and disaster preparedness plans in place is crucial for effectively responding to medical crises and minimising complications. Adequate emergency care capabilities and infrastructure can help reduce the financial burden on national budgets, make optimal use of available human resources, and alleviate psychological distress for patients and their loved ones during times of medical emergency. Standardised emergency packages, regular staff training, ongoing quality monitoring, and engagement of leadership around emergency care could help   improve emergency response capabilities  and ultimately enhance patient outcomes.

## Supplementary Information

Below is the link to the electronic supplementary material.

Supplementary file1 (DOCX 50 KB)
