# Supplementary material for: Ecology of emergency care in lower-tier healthcare providers in Ghana: an empirical data-driven Bayesian network analytical approach
Source: Intern Emerg Med. 2024 Apr 29;19(8):1–13. doi: 10.1007/s11739-024-03607-6 (PMC11582174; doi:10.1007/s11739-024-03607-6)
Supplement: Supplementary file 1 — Supplementary file1 (DOCX 50 KB) [file 11739_2024_3607_MOESM1_ESM.docx]

Appendix S1: Standardised Health Facilities Emergency Preparedness Assessment Tool (HeFEPAT)

**INSTRUCTIONS FOR SCORING**

1. **Assess by Observation, Interviewing, and Reviewing of Methods and Documents.**
2. **In case of two-level scale, 1 represents ‘Yes’ and 0 represents ‘No’. For four level scale, 0 represents ‘non-available’, 1 represents ‘in-complete (1%-49%)’ 2 represents ‘fairly-complete (50%-99%)’ and 3 represents ‘complete (100%)’, where complete is equivalent to fully compliant, fully available, or fully appropriate.**

**NAME OF FACILITY……………………………………………………**

**TYPE OF FACILITY ……………………………………………………**

**DATE OF VISIT ……………………………………………………………**

|  | **Practitioner-in-Charge** |
| --- | --- |
|  | **Name :** |
|  | **Qualification/Degree:** |
|  | **Profession :** |
|  | **Years of Post Basic Qualification:** |
|  | **Telephone Number:** |
|  | **Ownership Type: NGO, Public, Private, CHAG, etc.,** |
|  | **Facility Location:** |
|  | **GPS Address (Ghana Post):** |
|  | **Town/City:** |
|  | **District/Municipality/Metropolis:** |
|  | **Region:** |
|  | **Postal Address:** |
|  | **Facility Telephone Number(s):** |
|  | **Email Address:** |
|  | **Year of establishment of the Facility:** |

| **ASSESSMENT TOOL** | | **Yes** | | **No** | | |
| --- | --- | --- | --- | --- | --- | --- |
| Practitioner-in-charge | 1. Availability |  | |  | | |
|  | 1. Equally qualified personnel |  | |  | | |
| Critical care personnel | 1. Trained critical care |  | |  | | |
| Emergency room or area | 1. Available |  | |  | | |
|  | 1. Dedicated bed |  | |  | | |
|  | 1. Oxygen with flowmeter |  | |  | | |
|  | 1. Defibrillator |  | |  | | |
|  | 1. Defibrillator protocols |  | |  | | |
| Cerebrovascular Accident | 1. Cerebrovascular Accident protocols |  | |  | | |
|  | 1. Cerebrovascular Accident medications (iv thrombolytic and mannitol infusion |  | |  | | |
| Diabetic emergency | 1. Diabetic emergency (DKA) protocols |  | |  | | |
|  | 1. Insulin and sliding scale |  | |  | | |
| Heart failure emergency | 1. Heart failure emergency protocols |  | |  | | |
|  | 1. Heart failure emergency medications (iv beta-blocker and iv diuretic) |  | |  | | |
| Road Traffic Accident | 1. Road Traffic Accident protocols |  | |  | | |
| Acute care | 1. Acute care protocols |  | |  | | |
| Acute Care |  | **0** | **1** | | **2** | **3** |
|  | 1. Knowledge on Acute care protocols |  |  | |  |  |
|  | 1. Demonstration of cardiopulmonary resuscitation |  |  | |  |  |

**Comment**

**……………………………………………………………………………………………………………………………………………………….**

**……………………………………………………………………………………………………………………………………………………….**

**……………………………………………………………………………………………………………………………………………………….**

Appendix S2: Conditional probability tables for the Bayesian Network models.

**Facility**

| Region | Facility | P(Facility) |
| --- | --- | --- |
| Ashanti | PH | 0.38 |
| G Accra | PH | 0.74 |
| Ashanti | Poly | 0.05 |
| G Accra | Poly | 0.005 |
| Ashanti | Clinic | 0.27 |
| G Accra | Clinic | 0.06 |
| Ashanti | HC | 0.16 |
| G Accra | HC | 0.08 |
| Ashanti | MH | 0.14 |
| G Accra | MH | 0.115 |

**Ownership**

| Facility | Owner | P(Owner) |
| --- | --- | --- |
| PH | Gov | 0.11 |
| PH | CHAG | 0.07 |
| PH | Private | 0.8 |
| PH | Quasi | 0.016 |
| PH | NGO | 0.004 |
| Poly | Gov | 1 |
| Poly | CHAG | 0 |
| Poly | Private | 0 |
| Poly | Quasi | 0 |
| Poly | NGO | 0 |
| Clinic | Gov | 0.07 |
| Clinic | CHAG | 0 |
| Clinic | Private | 0.93 |
| Clinic | Quasi | 0 |
| Clinic | NGO | 0 |
| HC | Gov | 0.78 |
| HC | CHAG | 0.05 |
| HC | Private | 0.17 |
| HC | Quasi | 0 |
| HC | NGO | 0 |
| MH | Gov | 0.05 |
| MH | CHAG | 0 |
| MH | Private | 0.95 |
| MH | Quasi | 0 |
| MH | NGO | 0 |

**Defibrillator protocols**

| D | DP | P(DP) |
| --- | --- | --- |
| Yes | Yes | 1 |
| Yes | No | 0 |
| No | Yes | 0.007 |
| No | No | 0.993 |

**Personnel in charge**

| Facility | In-charge | P(Owner) |
| --- | --- | --- |
| PH | MO | 1 |
| PH | Midwife | 0 |
| PH | Nurse | 0 |
| PH | PA | 0 |
| Poly | MO | 1 |
| Poly | Midwife | 0 |
| Poly | Nurse | 0 |
| Poly | PA | 0 |
| Clinic | MO | 0.5 |
| Clinic | Midwife | 0.5 |
| Clinic | Nurse | 0 |
| Clinic | PA | 0 |
| HC | MO | 0.05 |
| HC | Midwife | 0.17 |
| HC | Nurse | 0.02 |
| HC | PA | 0.76 |
| MH | MO | 0 |
| MH | Midwife | 1 |
| MH | Nurse | 0 |
| MH | PA | 0 |

**Cerebrovascular accident protocols**

| CEM | CAP | P(CAP) |
| --- | --- | --- |
| Yes | Yes | 1 |
| Yes | No | 0 |
| No | Yes | 0.108 |
| No | No | 0.892 |

**Diabetic emergency protocols**

| D | DP | P(DP) |
| --- | --- | --- |
| Yes | Yes | 0.962 |
| Yes | No | 0.038 |
| No | Yes | 0.02 |
| No | No | 0.98 |

**Heart failure emergency protocols**

| D | DP | P(DP) |
| --- | --- | --- |
| Yes | Yes | 1 |
| Yes | No | 0 |
| No | Yes | 0.06 |
| No | No | 0.94 |

**Acute care protocols**

| Facility | Owner | ACP | P(ACP) |
| --- | --- | --- | --- |
| PH | Gov | Yes | 0.793 |
| PH | CHAG | Yes | 0.667 |
| PH | Private | Yes | 0.975 |
| PH | Quasi | Yes | 1 |
| PH | NGO | Yes | 1 |
| Poly | Gov | Yes | 1 |
| Poly | CHAG | Yes | 0 |
| Poly | Private | Yes | 0 |
| Poly | Quasi | Yes | 0 |
| Poly | NGO | Yes | 0 |
| Clinic | Gov | Yes | 0 |
| Clinic | CHAG | Yes | 0 |
| Clinic | Private | Yes | 0.397 |
| Clinic | Quasi | Yes | 0 |
| Clinic | NGO | Yes | 0 |
| HC | Gov | Yes | 0.174 |
| HC | CHAG | Yes | 0 |
| HC | Private | Yes | 0.1 |
| HC | Quasi | Yes | 0 |
| HC | NGO | Yes | 0 |
| MH | Gov | Yes | 0 |
| MH | CHAG | Yes | 0 |
| MH | Private | Yes | 0.286 |
| MH | Quasi | Yes | 0 |
| MH | NGO | Yes | 0 |
| PH | Gov | No | 0.207 |
| PH | CHAG | No | 0.333 |
| PH | Private | No | 0.025 |
| PH | Quasi | No | 0 |
| PH | NGO | No | 0 |
| Poly | Gov | No | 0 |
| Poly | CHAG | No | 1 |
| Poly | Private | No | 1 |
| Poly | Quasi | No | 1 |
| Poly | NGO | No | 1 |
| Clinic | Gov | No | 1 |
| Clinic | CHAG | No | 1 |
| Clinic | Private | No | 0.603 |
| Clinic | Quasi | No | 1 |
| Clinic | NGO | No | 1 |
| HC | Gov | No | 0.826 |
| HC | CHAG | No | 1 |
| HC | Private | No | 0.9 |
| HC | Quasi | No | 1 |
| HC | NGO | No | 1 |
| MH | Gov | No | 1 |
| MH | CHAG | No | 1 |
| MH | Private | No | 0.714 |
| MH | Quasi | No | 1 |
| MH | NGO | No | 1 |

**Critical care personnel**

| Owner | In-charge | CCP | P(CCP) |
| --- | --- | --- | --- |
| Gov | MO | Yes | 0.838 |
| Gov | Midwife | Yes | 0 |
| Gov | Nurse | Yes | 0 |
| Gov | PA | Yes | 0 |
| CHAG | MO | Yes | 0.333 |
| CHAG | Midwife | Yes | 0.5 |
| CHAG | Nurse | Yes | 0 |
| CHAG | PA | Yes | 0 |
| Private | MO | Yes | 0.214 |
| Private | Midwife | Yes | 0 |
| Private | Nurse | Yes | 0 |
| Private | PA | Yes | 0 |
| Quasi | MO | Yes | 1 |
| Quasi | Midwife | Yes | 0 |
| Quasi | Nurse | Yes | 0 |
| Quasi | PA | Yes | 0 |
| NGO | MO | Yes | 1 |
| NGO | Midwife | Yes | 0 |
| NGO | Nurse | Yes | 0 |
| NGO | PA | Yes | 0 |
| Gov | MO | No | 0.162 |
| Gov | Midwife | No | 1 |
| Gov | Nurse | No | 1 |
| Gov | PA | No | 1 |
| CHAG | MO | No | 0.667 |
| CHAG | Midwife | No | 0.5 |
| CHAG | Nurse | No | 1 |
| CHAG | PA | No | 1 |
| Private | MO | No | 0.786 |
| Private | Midwife | No | 1 |
| Private | Nurse | No | 1 |
| Private | PA | No | 1 |
| Quasi | MO | No | 0 |
| Quasi | Midwife | No | 1 |
| Quasi | Nurse | No | 1 |
| Quasi | PA | No | 1 |
| NGO | MO | No | 0 |
| NGO | Midwife | No | 1 |
| NGO | Nurse | No | 1 |
| NGO | PA | No | 1 |

**Emergency area**

| Owner | In-charge | EA | P(EA) |
| --- | --- | --- | --- |
| Gov | MO | Yes | 1 |
| Gov | Midwife | Yes | 0 |
| Gov | Nurse | Yes | 0 |
| Gov | PA | Yes | 0.031 |
| CHAG | MO | Yes | 1 |
| CHAG | Midwife | Yes | 0 |
| CHAG | Nurse | Yes | 0 |
| CHAG | PA | Yes | 0 |
| Private | MO | Yes | 0.848 |
| Private | Midwife | Yes | 0.054 |
| Private | Nurse | Yes | 0 |
| Private | PA | Yes | 0 |
| Quasi | MO | Yes | 1 |
| Quasi | Midwife | Yes | 0 |
| Quasi | Nurse | Yes | 0 |
| Quasi | PA | Yes | 0 |
| NGO | MO | Yes | 1 |
| NGO | Midwife | Yes | 0 |
| NGO | Nurse | Yes | 0 |
| NGO | PA | Yes | 0 |
| Gov | MO | No | 0 |
| Gov | Midwife | No | 1 |
| Gov | Nurse | No | 1 |
| Gov | PA | No | 0.969 |
| CHAG | MO | No | 0 |
| CHAG | Midwife | No | 1 |
| CHAG | Nurse | No | 1 |
| CHAG | PA | No | 1 |
| Private | MO | No | 0.152 |
| Private | Midwife | No | 0.946 |
| Private | Nurse | No | 1 |
| Private | PA | No | 1 |
| Quasi | MO | No | 0 |
| Quasi | Midwife | No | 1 |
| Quasi | Nurse | No | 1 |
| Quasi | PA | No | 1 |
| NGO | MO | No | 0 |
| NGO | Midwife | No | 1 |
| NGO | Nurse | No | 1 |
| NGO | PA | No | 1 |

**Oxygen wit flowmeter**

| Owner | In-charge | EA | P(EA) |
| --- | --- | --- | --- |
| Gov | MO | Yes | 1 |
| Gov | Midwife | Yes | 1 |
| Gov | Nurse | Yes | 0 |
| Gov | PA | Yes | 0.844 |
| CHAG | MO | Yes | 1 |
| CHAG | Midwife | Yes | 0 |
| CHAG | Nurse | Yes | 0 |
| CHAG | PA | Yes | 1 |
| Private | MO | Yes | 0.953 |
| Private | Midwife | Yes | 0.321 |
| Private | Nurse | Yes | 0 |
| Private | PA | Yes | 1 |
| Quasi | MO | Yes | 1 |
| Quasi | Midwife | Yes | 0 |
| Quasi | Nurse | Yes | 0 |
| Quasi | PA | Yes | 0 |
| NGO | MO | Yes | 1 |
| NGO | Midwife | Yes | 0 |
| NGO | Nurse | Yes | 0 |
| NGO | PA | Yes | 0 |
| Gov | MO | No | 0 |
| Gov | Midwife | No | 0 |
| Gov | Nurse | No | 1 |
| Gov | PA | No | 0.156 |
| CHAG | MO | No | 0 |
| CHAG | Midwife | No | 1 |
| CHAG | Nurse | No | 1 |
| CHAG | PA | No | 0 |
| Private | MO | No | 0.047 |
| Private | Midwife | No | 0.679 |
| Private | Nurse | No | 1 |
| Private | PA | No | 0 |
| Quasi | MO | No | 0 |
| Quasi | Midwife | No | 1 |
| Quasi | Nurse | No | 1 |
| Quasi | PA | No | 1 |
| NGO | MO | No | 0 |
| NGO | Midwife | No | 1 |
| NGO | Nurse | No | 1 |
| NGO | PA | No | 1 |

**Defibrillator**

| Owner | In-charge | D | P(D) |
| --- | --- | --- | --- |
| Gov | MO | Yes | 0 |
| Gov | Midwife | Yes | 0 |
| Gov | Nurse | Yes | 0 |
| Gov | PA | Yes | 0 |
| CHAG | MO | Yes | 0 |
| CHAG | Midwife | Yes | 0 |
| CHAG | Nurse | Yes | 0 |
| CHAG | PA | Yes | 0 |
| Private | MO | Yes | 0.156 |
| Private | Midwife | Yes | 0 |
| Private | Nurse | Yes | 0 |
| Private | PA | Yes | 0 |
| Quasi | MO | Yes | 1 |
| Quasi | Midwife | Yes | 0 |
| Quasi | Nurse | Yes | 0 |
| Quasi | PA | Yes | 0 |
| NGO | MO | Yes | 0 |
| NGO | Midwife | Yes | 0 |
| NGO | Nurse | Yes | 0 |
| NGO | PA | Yes | 0 |
| Gov | MO | No | 1 |
| Gov | Midwife | No | 1 |
| Gov | Nurse | No | 1 |
| Gov | PA | No | 1 |
| CHAG | MO | No | 1 |
| CHAG | Midwife | No | 1 |
| CHAG | Nurse | No | 1 |
| CHAG | PA | No | 1 |
| Private | MO | No | 0.844 |
| Private | Midwife | No | 1 |
| Private | Nurse | No | 1 |
| Private | PA | No | 1 |
| Quasi | MO | No | 0 |
| Quasi | Midwife | No | 1 |
| Quasi | Nurse | No | 1 |
| Quasi | PA | No | 1 |
| NGO | MO | No | 1 |
| NGO | Midwife | No | 1 |
| NGO | Nurse | No | 1 |
| NGO | PA | No | 1 |

**Cerebrovascular emergency medication**

| Owner | In-charge | CEM | P(CEM) |
| --- | --- | --- | --- |
| Gov | MO | Yes | 0.649 |
| Gov | Midwife | Yes | 0 |
| Gov | Nurse | Yes | 0 |
| Gov | PA | Yes | 0 |
| CHAG | MO | Yes | 0.167 |
| CHAG | Midwife | Yes | 0 |
| CHAG | Nurse | Yes | 0 |
| CHAG | PA | Yes | 0 |
| Private | MO | Yes | 0.214 |
| Private | Midwife | Yes | 0 |
| Private | Nurse | Yes | 0 |
| Private | PA | Yes | 0 |
| Quasi | MO | Yes | 1 |
| Quasi | Midwife | Yes | 0 |
| Quasi | Nurse | Yes | 0 |
| Quasi | PA | Yes | 0 |
| NGO | MO | Yes | 0 |
| NGO | Midwife | Yes | 0 |
| NGO | Nurse | Yes | 0 |
| NGO | PA | Yes | 0 |
| Gov | MO | No | 0.351 |
| Gov | Midwife | No | 1 |
| Gov | Nurse | No | 1 |
| Gov | PA | No | 1 |
| CHAG | MO | No | 0.833 |
| CHAG | Midwife | No | 1 |
| CHAG | Nurse | No | 1 |
| CHAG | PA | No | 1 |
| Private | MO | No | 0.786 |
| Private | Midwife | No | 1 |
| Private | Nurse | No | 1 |
| Private | PA | No | 1 |
| Quasi | MO | No | 0 |
| Quasi | Midwife | No | 1 |
| Quasi | Nurse | No | 1 |
| Quasi | PA | No | 1 |
| NGO | MO | No | 1 |
| NGO | Midwife | No | 1 |
| NGO | Nurse | No | 1 |
| NGO | PA | No | 1 |

**Diabetic emergency medication**

| Owner | In-charge | DEM | P(DEM) |
| --- | --- | --- | --- |
| Gov | MO | Yes | 1 |
| Gov | Midwife | Yes | 0.158 |
| Gov | Nurse | Yes | 0 |
| Gov | PA | Yes | 0.281 |
| CHAG | MO | Yes | 1 |
| CHAG | Midwife | Yes | 0 |
| CHAG | Nurse | Yes | 0 |
| CHAG | PA | Yes | 0 |
| Private | MO | Yes | 0.83 |
| Private | Midwife | Yes | 0.054 |
| Private | Nurse | Yes | 0 |
| Private | PA | Yes | 0.071 |
| Quasi | MO | Yes | 1 |
| Quasi | Midwife | Yes | 0 |
| Quasi | Nurse | Yes | 0 |
| Quasi | PA | Yes | 0 |
| NGO | MO | Yes | 0 |
| NGO | Midwife | Yes | 0 |
| NGO | Nurse | Yes | 0 |
| NGO | PA | Yes | 0 |
| Gov | MO | No | 0 |
| Gov | Midwife | No | 0.842 |
| Gov | Nurse | No | 1 |
| Gov | PA | No | 0.719 |
| CHAG | MO | No | 0 |
| CHAG | Midwife | No | 1 |
| CHAG | Nurse | No | 1 |
| CHAG | PA | No | 1 |
| Private | MO | No | 0.17 |
| Private | Midwife | No | 0.946 |
| Private | Nurse | No | 1 |
| Private | PA | No | 0.929 |
| Quasi | MO | No | 0 |
| Quasi | Midwife | No | 1 |
| Quasi | Nurse | No | 1 |
| Quasi | PA | No | 1 |
| NGO | MO | No | 1 |
| NGO | Midwife | No | 1 |
| NGO | Nurse | No | 1 |
| NGO | PA | No | 1 |

**Heat failure emergency medication**

| Owner | In-charge | HEM | P(HEM) |
| --- | --- | --- | --- |
| Gov | MO | Yes | 0.541 |
| Gov | Midwife | Yes | 0 |
| Gov | Nurse | Yes | 0 |
| Gov | PA | Yes | 0 |
| CHAG | MO | Yes | 0.333 |
| CHAG | Midwife | Yes | 0 |
| CHAG | Nurse | Yes | 0 |
| CHAG | PA | Yes | 0 |
| Private | MO | Yes | 0.152 |
| Private | Midwife | Yes | 0 |
| Private | Nurse | Yes | 0 |
| Private | PA | Yes | 0 |
| Quasi | MO | Yes | 0 |
| Quasi | Midwife | Yes | 0 |
| Quasi | Nurse | Yes | 0 |
| Quasi | PA | Yes | 0 |
| NGO | MO | Yes | 0 |
| NGO | Midwife | Yes | 0 |
| NGO | Nurse | Yes | 0 |
| NGO | PA | Yes | 0 |
| Gov | MO | No | 0.459 |
| Gov | Midwife | No | 1 |
| Gov | Nurse | No | 1 |
| Gov | PA | No | 1 |
| CHAG | MO | No | 0.667 |
| CHAG | Midwife | No | 1 |
| CHAG | Nurse | No | 1 |
| CHAG | PA | No | 1 |
| Private | MO | No | 0.848 |
| Private | Midwife | No | 1 |
| Private | Nurse | No | 1 |
| Private | PA | No | 1 |
| Quasi | MO | No | 1 |
| Quasi | Midwife | No | 1 |
| Quasi | Nurse | No | 1 |
| Quasi | PA | No | 1 |
| NGO | MO | No | 1 |
| NGO | Midwife | No | 1 |
| NGO | Nurse | No | 1 |
| NGO | PA | No | 1 |

**Road traffic accident protocols**

| Facility | Owner | RAP | P(RAP) |
| --- | --- | --- | --- |
| PH | Gov | Yes | 0.207 |
| PH | CHAG | Yes | 0 |
| PH | Private | Yes | 0 |
| PH | Quasi | Yes | 1 |
| PH | NGO | Yes | 0 |
| Poly | Gov | Yes | 0 |
| Poly | CHAG | Yes | 0 |
| Poly | Private | Yes | 0 |
| Poly | Quasi | Yes | 0 |
| Poly | NGO | Yes | 0 |
| Clinic | Gov | Yes | 0 |
| Clinic | CHAG | Yes | 0 |
| Clinic | Private | Yes | 0 |
| Clinic | Quasi | Yes | 0 |
| Clinic | NGO | Yes | 0 |
| HC | Gov | Yes | 0 |
| HC | CHAG | Yes | 0 |
| HC | Private | Yes | 0 |
| HC | Quasi | Yes | 0 |
| HC | NGO | Yes | 0 |
| MH | Gov | Yes | 0 |
| MH | CHAG | Yes | 0 |
| MH | Private | Yes | 0 |
| MH | Quasi | Yes | 0 |
| MH | NGO | Yes | 0 |
| PH | Gov | No | 0.793 |
| PH | CHAG | No | 1 |
| PH | Private | No | 1 |
| PH | Quasi | No | 0 |
| PH | NGO | No | 1 |
| Poly | Gov | No | 1 |
| Poly | CHAG | No | 1 |
| Poly | Private | No | 1 |
| Poly | Quasi | No | 1 |
| Poly | NGO | No | 1 |
| Clinic | Gov | No | 1 |
| Clinic | CHAG | No | 1 |
| Clinic | Private | No | 1 |
| Clinic | Quasi | No | 1 |
| Clinic | NGO | No | 1 |
| HC | Gov | No | 1 |
| HC | CHAG | No | 1 |
| HC | Private | No | 1 |
| HC | Quasi | No | 1 |
| HC | NGO | No | 1 |
| MH | Gov | No | 1 |
| MH | CHAG | No | 1 |
| MH | Private | No | 1 |
| MH | Quasi | No | 1 |
| MH | NGO | No | 1 |
